# Neuromarketing Applied to Educational Toy Packaging

**DOI:** 10.3389/fpsyg.2020.02077

**Published:** 2020-08-25

**Authors:** David Juarez, Victoria Tur-Viñes, Ana Mengual

**Affiliations:** ^1^Institute of Technology of Material, Universitat Politècnica de València, València, Spain; ^2^Department of Communication and Social Psychology, University of Alicante, Alicante, Spain; ^3^Department of Business Organization, Universitat Politècnica de València, València, Spain

**Keywords:** psychology, marketing, neuromarketing, educational toy, packaging, eye tracking, galvanic skin response

## Abstract

This research work comes in response to the question of which aspects are more relevant for consumers when purchasing educational toys as opposed to other toys that are focused solely on leisure. This empirical research focuses on an educational toy distributed in Spain by the Educa brand (Conector family, reference “I learn English”), which is the product best-selling product of its brand in this area, and analyses how consumers make decisions concerning this product in relation to other products designed by competitors. The research looks into customer reactions while looking at these products, measuring brain activity generated by different aspects of product design and its influence on choice. The aim of the present study was to propose a model that optimizes the design of educational toy packaging. Through the use of neuromarketing techniques –attention through eye tracking, and emotion using galvanic skin response– as well as qualitative research techniques, the objective of this research is to determine the motivations in the processes of buying educational toys. The packaging design elements analyzed are brand, product family, toy name, recommended age, game image, number of questions/topics, and additional texts. The results suggest that the most important elements are the graphic details of the packaging, obtaining a perception of a higher educational level as more questions are addressed by the game. The simultaneous combination of qualitative techniques monitored with galvanic skin response (neuro-qualitative study) allows additional conclusions to be aligned with the end user of the product, including a prominent social component when the product is purchased as a gift for a third party.

## Introduction

The toy is that creation, handmade or industrial, designed and/or produced to stimulate and accompany the game (Espinosa, 2018). Any person, minor or adult, with more or less sophistication, can turn an object into play material. Through the toy we can stimulate imagination, creativity, movement, language, memory, etc. of children, attending to their needs, age and concerns. In this way, the game turns children into protagonists, enhancing any positive aspect of their personality (AIJU, 2019): motivating them to improve or to express their feelings, awakening their curiosity or their ability to laugh and imagine, providing them with learning and decisive experiences for their healthy growth and training as a person. Game and toys offer children the possibility of representing the world around them, as well as the social values that support it, through imitation of what they see and live in their daily lives.

Design defines objects and establishes approximations (Bloch, 1995), since it is a creative act that works with the intangible to create meaning at different cultural levels (AEFJ, 2019). For this reason, design requires theoretical foundations that in practice become meanings (Starks, 2014) and thus, the object-language is established. There is a risk of standardization, a concept that allows the designer to operate without compromising sensitivity and creativity. Moreover, the user interrelates it with the environment and previous experiences (Martínez-Sanz, 2012).

The toy can be considered to be a cultural product, occupying a promoting role to reinforce real life conceptions AIJU (2019). The toys are usually miniature replicas of real objects, and as such, the product does not come to life until it manifests itself in a playful act, when it arouses concern in the infant. The toy has an extensive weight of meaning within it, since the signs used in each design are appreciated in a unique way and promote their interpretation. Through the toy, children can represent images, characters and scenes from the real world and interact with their fantasies or other children (Brunner et al., 2009). It is an object that promotes physical and social competence and also, through the manipulation of the object, the child explores its properties to better understand their world. Similarly, the child reinforces his self-image, manifests feelings, fears, and concerns, and it is a way to resolve conflicts (Espinosa, 2018). It also exercises physically and mentally, as it stimulates the imagination. Adults are usually the first to set the trend in consumption (when selecting toys for newborns). Packaging is a fundamental part of the product, because in addition to containing, protecting and preserving the product, it allows it to reach the final consumer in optimal conditions, and more importantly is a valuable tool for promotion and sale.

Packaging is defined by the American Marketing Association (AMA), an international organization promoting marketing professionals, as follows: “Container used to protect, promote, transport, and/or identify a product. Packaging may vary from plastic wrap to a steel or wooden box or a drum. It can be primary (contains the product), secondary (contains one or more primary packages) or tertiary (contains one or more secondary packages)” (American Marketing Association, 2013).

Packaging, as an element, is designed and has become a useful means not only to protect the product, but also has a visual function (Nancarrow et al., 1998). It has been considered a silent communicator (Tur-Viñes et al., 2014). In this sense, Lamb (Lamb, 2008) maintains that a package must fulfill three functions:

– Content and protection of products. Packaging protects items from breakage, evaporation, spills, deterioration, light, heat, cold, contamination and other conditions.

Product promotion. A packaging differentiates a product from its competitors and can associate a new article with a family of products from the same manufacturer. Packaging uses designs, colors, shapes and materials with the intention of influencing consumer perception and purchasing behavior (Silayoi and Speece, 2007; Orth et al., 2010).

– Ease of storage, use, and disposal. Wholesalers and retailers prefer displays that are easy to ship, store and place on shelves. They also like packaging that protects products, prevents deterioration or breakage, and extends the life of products on shelves.

The packaging in the promotion of the product refers to the visual language, whose components are established based on chromatic, photographic, typographic and morphological codes (Azad et al., 2014). Thus, it also uses visual grammar as a way of organizing signifiers and meanings (Zhang et al., 2018). The form, as a basic visual element, gains significance when used together (Vilchis, 2008). Obviously, visual elements (Reimann et al., 2010) are used in the visual language to construct messages. These objects occupy a certain space, with a series of internal and external characteristics, “physical and material qualities such as color, texture, contour, and the arrangement or configuration of specific natural characteristics,” thus configuring the spatial aspects of appearance. He also affirms that forms can be understood as a structure or concept, therefore, he divides forms into real (where what is important is the appearance, which only depends on the object) and apparent (which is the active form, which depends on the circumstance). In this way, it can be affirmed that “angular objects are more effective in attracting attention and provoking ideas (Vecchiato and Roveda, 2010); curvilinear objects are more effective in triggering a positive emotional and aesthetic response” (Lidwell et al., 2010).

Package design has become a key marketing tool (Krishna et al., 2017), with many implications for the multi-sensory customer experience (Martínez-Ruiz et al., 2017), making it a very powerful tool for the commercial arena (Chen, 2014). It emphasizes the mental image of the product transmitted to the consumer (Alhamdi, 2020), confirming its role in the consumer’s attraction to design, color, size and shape (Salem, 2018). It is a decision factor in a potential purchase, with a relationship between perceived authenticity and product preference (Filaretova et al., 2017). Some companies use Limited Edition Packaging (LEP), used as a product shortage tactic, to create a limited supply (Dörnyei, 2020). Consequently, packaging can contribute as a competitive advantage in business strategy, beyond its function with marketing logistics (Rundh, 2005).

The toy sector is increasingly concerned about the packaging that surrounds toys (Soluciones-Packaging, 2016). Packaging plays a fundamental role in toys, because in addition to protecting products, many of them delicate and with small pieces, packaging plays a fundamental role in communicating their value and philosophy to parents and children (Lawrence et al., 2015). The packaging of toys must be efficient, cost-effective and must facilitate, as much as possible, both the protection of the product that it contains and the transport from the point of manufacture to the sales center, as well as presenting an attractive display on the shelves.

Although the child is already seduced by television advertisements (Medrano et al., 2016) or by friends in their environment, having a striking packaging which is full of creativity helps when choosing the product (Bloch, 1995) whether it be from the toy catalog or from the shelves of toy stores and department stores. In fact, depending on the type of toy, the ideal situation is one in which the packaging is open so that it can be touched and seen by children during their visits to stores, or even that it has an interactive point so that it has one more attachment point with potential buyers. In addition, it must be borne in mind that the packaging must be child-friendly, since, although many parents help their children to open gifts, the packaging of toys must be designed so that the smallest children can open them quickly.

Finally, many younger children play more with the boxes that wrap the gifts, than with the toys that they contain. Hence the importance of creating a different and creative packaging for toys, packaging that is part of the toy itself and that helps to enhance the illusion of the game.

When choosing a game or toy, it must also be considered that it transmits certain social values, promoting specific ways of understanding and relating to society (Espinosa, 2018). The educational toy stimulates intellectual development, through reasoning, attention, imagination and creativity or mastery of language. They are an attractive resource to reinforce children’s formal content learning, such as numbers and letters. An educational toy will allow values such as respect and tolerance toward people, norms and rules to be acquired and socialization to be promoted (Luévano Torres, 2013).

The game is a tool through which many questions can be explained and answered (AIJU, 2019). Examples of educational games are those such as: simple scientific experiments, puzzles, creative games, games that help to learn geography, history, science, etc. or interactive games on electronic devices (book-games, math and reading games, music games, memory and logic games, pattern recognition, games that work on social and emotional skills, applications that help you learn geography, history, science, etc., and the electronic version of puzzles or creative games).

The educational toy has a different utility approach than other toys, where different variables are work (Lăzăroiu et al., 2017), apart from leisure, so it is interesting to know the information processing in purchasing decision making.

## Materials and Methods

The aim of this research is to determine, through neuromarketing techniques, the cognitive perception that Spanish parents, between 35 and 45 years old, with children between the ages of 4 and 8, have toward educational toys appropriate to the age of their children. The level of learning is linked to the amount of knowledge contributed by the game and the influence on brand marketing. To do this, we used neuromarketing techniques which allowed us to analyze the attention of the subjects to the stimuli (eye tracking) and the emotional intensity experienced (Galvanic Skin Response).

### Objectives

This research work helps answer the question of which aspects are more relevant for consumers in purchasing educational toys, which will obviously be quite different from products more focused simply on leisure. This empirical research focuses on an educational toy distributed in Spain by Educa brand (Conector family, reference “I learn English”), which is the brand’s best seller in this market area, and analyses how consumers make decisions regarding their choice in relation to other products designed by competitors. The study looks at customer reactions when looking at the products, measuring brain activity generated by different aspects of product design and its influence on choice.

The main objective of the research is to analyze the attention of parents toward the projection of images of the packaging of educational toys aimed at children with an age range between 4 and 8 years, and the emotional intensity registered when considering the purchase. The specific objectives are as follows:

– Analyze the attention generated by the different elements of the packaging of an educational toy (comparison with two similar products of competing brands) between parents.
– Verify the differences in care of each element of the package compared to its size and layout, according to the corresponding brand.
– Determine what differences there are between parents, according to gender.
– Analyze the emotional intensity generated in the parents, according to the purchase intention.

### Research Instrument

One of the most interesting questions in traditional research is concerning future behavior intentions (Hammou et al., 2013). The problem is that human being build the future by imagining what has been done in the past, in a similar circumstance (Fugate, 2008), or by trying to estimate what the feelings or emotions about the event will be. There is the problem of obtaining the correct answers to the questions posed to consumers in traditional market research (Malhotra et al., 2007). This is known as “bias” and has been studied and documented by psychologists, differentiating between conscious and unconscious “bias.” When consumers are asked about past experiences regarding purchasing products, or how they relate to brands (Morin et al., 2016), it is possible to get incorrect answers because what is remembered, many times, is partial or has been distorted based on different factors. In the end, the brain constructs reality with the partial information it has and fills the gaps in memory with information that seems coherent (Crone and Ridderinkhof, 2011).

As a result of combining neuroscience with marketing, neuromarketing is emerging as a relatively new research discipline (Morin, 2011). Advances in technology allow this new field to go beyond traditional quantitative and qualitative research tools, and focus on consumers’ brain reactions to marketing stimuli (Reimann et al., 2011). It is new discipline that applies the knowledge of the latest brain research to the world of management (Madan, 2010). These theories allow designers to combine the best of marketing with the best of sales, since both processes have the main objective of motivating people to make purchasing decisions (Ohme et al., 2010).

The main objective of marketing is to link products and people (Ariely and Jones, 2008). Neuromarketing research aims to connect activity in the neural system with consumer behavior, and has a wide variety of applications for brands, products, packaging, advertising or marketing for stores, to be able to determine the intention to buy, level of novelty, awareness or emotions generated. Although the collection of neuroimaging data involves a quantitative approach, which measures our brain activity in numbers, neuromarketing research seems to have common aspects also with the qualitative side of the research. Butler (2008) proposes a neuromarketing research model that connects marketing researchers, practitioners, and other stakeholders, and states that more research is needed to establish its academic relevance.

As neuromarketing is a fairly young discipline, the theoretical, empirical and practical field is still in development (Garcia and Saad, 2008). Theoretical research in neuromarketing is based on neuroscience, and neuroimaging techniques are used in this emerging field in order to test the hypothesis, improve existing knowledge, or to test the effect of marketing stimuli on the brain of the consumer (Karmarkar and Plassmann, 2017). The research established that patterns of brain activity are closely related to behavior and cognition (Alwitt and Mitchell, 1985).

According to the classic assumption, consumers, in their decision-making process, take into account all the possible alternatives in the market and select the one that maximizes «marginal utility». This assumption is no longer valid, as stated by Kahneman (2012), psychologist and Nobel Prize in Economics in 2002, whose works are developed in the line of decision-making in uncertain environments, and the use of heuristics and shortcuts mental.

Consequently, the research technique that has been used in this study is neuromarketing. Its purpose is to measure the cognitive processing of the stimuli designed in the packaging of educational toys. The neurodata (Loyola et al., 2016) used is based on eye tracking, a biometric technique that records the visual attention of the subjects based on their eye movements (Duchowski, 2017; Mañas-Viniegra et al., 2020), which are directed toward areas that are of interest to the subject, also known as areas of interest (AOI), and the GSR, also known as electrodermal activity (EDA), collects changes in the state of emotional excitement, which influence the cognitive perception of stimuli (Critchley, 2002; Mañas-Viniegra et al., 2020). When the subjects turn their attention to a stimulus, it is registered through the eye tracking system and initiates cognitive and affective processing, which produces an influence on the preferences of the audience or the consumer (Bornstein and D’agostino, 1992; Pieters et al., 2002; Goodrich, 2011; Mañas-Viniegra et al., 2020). Neuromarketing and neuroeconomics have made it possible to confirm certain traditional marketing postulates such as the effectiveness of emotional advertising and, above all, to destroy the classic paradigm of “rational behavior” of the consumer (Álvarez-del-Blanco, 2011).

### Sample

In the present research, the sample consisted of men and women, according to the indications of the manufacturer Educa, from current consumer data. A total of 30 people (33% men and 66% women) participated randomly and voluntarily as study subjects after meeting the requirements of being parents, aged between 35 and 45 with children of ages between 4 and 8 years old. The percentage of men and women was adjusted to the manufacturers’ indications, since this is the distribution of percentages, by gender, of their target. Alicante (Spain) was chosen for the sample due to its status as a provincial capital. The sample size (consisting of 10 men and 20 women) was adequate for a neuromarketing study (Cuesta-Cambra et al., 2017). After carrying out the empirical study, five users (all belonging to the female gender) were discarded, leaving 25 users (10 men and 15 women).

### Data Collection and Analysis

The research phase with packaging was performed using the eye tracker model Gazepoint GP3HD, with a 150 Hz sampling rate. For data collection, Gazepoint Analysis UX Edition v.5.3.0 software was used. The Shimmer3 GSR + model was used to record electrodermal activity, using the ConsensysPRO software, v.1.6, for data collection. Subjects were exposed to two random stimuli (packaging image) from the manufacturers Educa and Diset. Each stimulus had a maximum time limit of 30 s – with 5 s of separation between stimuli – to prioritize the areas of interest that captured the most attention and emotion. This was explained to the participants, since it is equivalent to a time similar to that spent on the shelf. These two stimuli, which were similar to each other, were selected to show comparable products and packaging.

**FIGURE 1:**
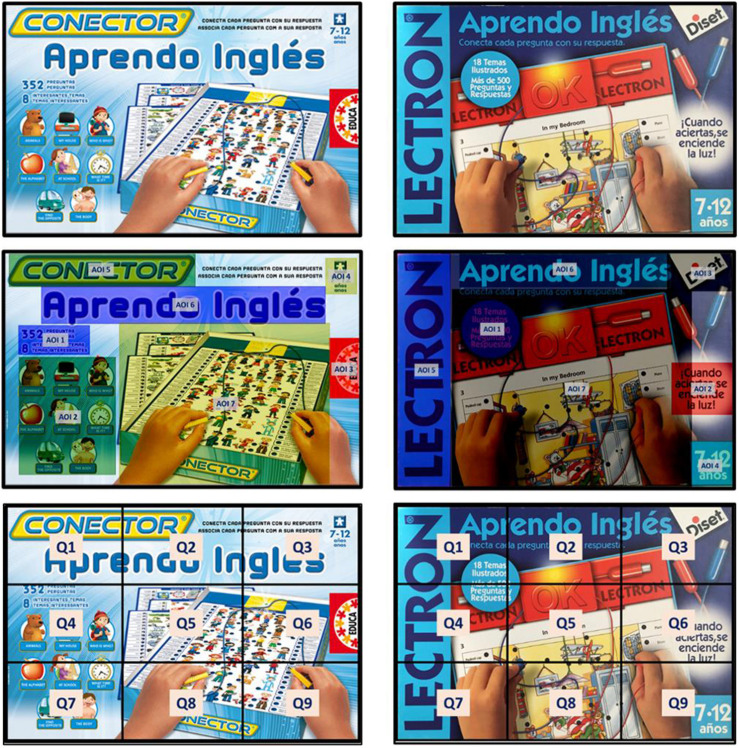
Packaging, areas of interest, and design quadrants. Source: Prepared by the authors.

The statistical analysis of the data was performed with the R software, v.3.6.3. The common elements (stimuli) between both packages were defined, as well as location quadrants (Figure 1 and Table 1). Subjects were exposed to two packages containing seven stimuli each, comparable to each other. The stimulus 02 of each brand is free (not equivalent). Each package had a maximum time limit of 30 s – with 3 s of separation between stimuli – to prioritize the areas of interest that captured the most attention (Añaños-Carrasco, 2015).

**TABLE 1:** Common stimuli by brand, location quadrant, and size ratio.

**Stimulus**				**Area ratio**	**Area (cm^2^)**	**Area (cm^2^)**
**number**	**Stimulus name**	**Brand**	**Quadrant**	**EDUCA = 1**	**EDUCA**	**DISET**
1	Number of questions/topics	EDUCA	Q4	1: 1.3	30.0	38.5
		DISET	Q1 + Q4			
2	Game topics	EDUCA	Q4 + Q7	1: 0.2	143.0	32.5
	Message	DISET	Q6			
3	Trademark	EDUCA	Q6	1: 1.4	19.5	28.0
		DISET	Q3			
4	Recommended age	EDUCA	Q3	1: 5.0	6.0	30.0
		DISET	Q9			
5	Product family	EDUCA	Q1 + Q2	1: 2.3	71.8	162.0
		DISET	Q1 + Q4 + Q7			
6	Product name	EDUCA	Q1 + Q2 + Q3	1: 0.7	98.0	63.8
		DISET	Q1 + Q2 + Q3			
7	Game picture	EDUCA	Q5 + Q6 + Q8 + Q9	1: 1.7	408.0	689.9
		DISET	Q2 + Q3 + Q4 + Q5 + Q6 + Q7 + Q8 + Q9			

**Source: Prepared by the authors.**

The independent variable was the sex of the participants, with a similar sociocultural profile in all of them, and determined by the main profile of the company’s target. The dependent variables were the level of attention and the emotional arousal peaks recorded in response to the observed stimuli.

Quantitative data analysis was used to evaluate the seconds that elapsed between the appearance of the stimulus and the first fixation, or the Time First Fixation (TFF), the number of eye fixations, or the Fixation Count (FC), and the total number of seconds of attention to each area of interest, or Total Fixation Duration (TFD). The qualitative evaluation was performed using thermal maps of the attention registered by the eye tracker.

Regarding the semi-structured in-depth interview, the interview protocol was designed to provide evidence of the experience of buying this category of toy. The interviews were carried out by the authors. All interviews were conducted face-to-face, recording the electrodermal activity. The application of neuromarketing to qualitative research allows a record of the arousal, or general physiological and psychological activation of the organism (Gould and Krane, 1992), experienced by the subject during an in-depth interview, neuro-qualitative study. All interviews were videotaped, transcribed, and analyzed.

Regarding the records of the GSR peaks, which can occur up to 3 s after the start of emotional activation, it was applied to determine emotional arousal during the in-depth interview.

The qualitative research phase (in-depth interviews) was monitored using the Shimmer3 GSR galvanic skin response model. ConsensysPRO v1.6.0 software was used for data collection.

## Results

### Comprehensive Analysis of Attention

The Kruskal – Wallis test was used to show that visual attention is greater in women than in men (Cuesta-Cambra et al., 2017). The following measurements were used: Average Fixation Duration (AFD), total number of fixations, and frequency of fixations per second, total time of fixations. Kruskal Wallis’ method has shown that the total number of fixations and the total time of fixations are greater, with significant differences for women than for men as can be seen in Table 2 (^∗^*p* < 0.5: ^∗∗^*p* < 0.1).

**TABLE 2:** Test of Kruskal Wallis (p* < 0.01).

	**Men**		**Woman**	
**Eye tracking metrics**	**EDUCA**	**DISET**	**EDUCA**	**DISET**
Average fixation duration (mls)	256	214	351*	311 *
Total number of fixations	956	987	1102*	1470*
Fixations per second	1.05	1.29	1.45*	1.98*
Total time of the fixations	774	756	989*	956*

**FIGURE 2:**
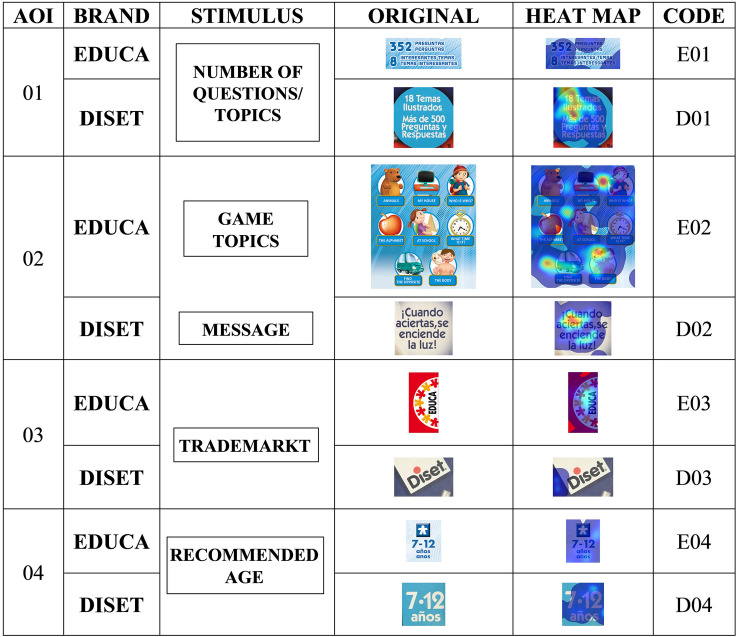
Heat maps of stimuli. Part 1. Relation to scale. Source: Prepared by the authors.

**FIGURE 3:**
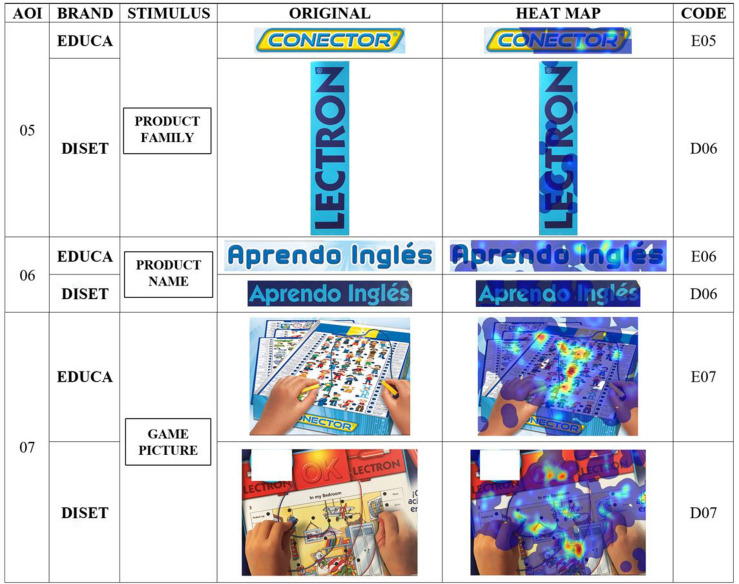
Heat maps of stimuli. Part 2. Relation to scale. Source: Prepared by the authors.

The heat maps produced by the attention of the participants on the different parts of the container, for both brands (Figures 2, 3), qualitatively reflect more intense attention on the graphic elements (drawings) and the information on the number of questions, and, to a lesser extent, on the brands or the name of the game.

In the first comprehensive quantitative analysis of attention that the entire group of subjects showed toward stimuli (Table 3), it was observed that the image of the game (AOI 7) attracted the highest percentage of attention of all the participants, with Diset the product paid most attention (D07 has values greater than 50%), both globally and by gender. As an interesting fact, it should be noted that this stimulus occupies 70% more area and is divided into eight quadrants, compared to four quadrants of the Educa equivalent. The stimulus 02 (themes of the game for Educa and operating message for Diset) is the second in percentage of attention, while Educa (E02) is the product with the highest percentage (global and by gender) highlighting that it is an image (500% greater in area) and occupies two quadrants (Q4 and Q7), compared to stimulus D02, which is a text and located in a single quadrant (Q6). Stimulus 01 (number of questions for both packages) reflects a high percentage of attention, with Diset (D01) as the product with the highest percentage (global and by gender) and highlighting that it is designed in a round shape, compared to that of Educa, which is rectangular (as well as being 30% bigger).

**TABLE 3:** Attention percentage of the total number of participants to each AOI.

	**Group**		**Men**		**Women**	
	**Attention rate (%)**		**Attention rate (%)**		**Attention rate (%)**	
**AOI**	**EDUCA**	**DISET**	**EDUCA**	**DISET**	**EDUCA**	**DISET**
AOI 1	5.97	12.75	5.33	12.01	6.50	13.31
AOI 2	16.80	4.12	15.28	3.38	18.09	4.64
AOI 3	1.47	1.97	1.63	1.67	1.31	2.15
AOI 4	2.26	6.94	3.64	6.80	1.49	7.05
AOI 5	2.45	4.14	3.07	4.63	1.97	3.76
AOI 6	6.66	5.20	7.49	6.23	5.91	4.54
AOI 7	26.17	54.03	28.41	55.85	24.26	52.60

**Separation by gender. Source: Prepared by the authors.**

Brand, recommended age, product family, and game name are the elements that receive the least percentage of attention.

### Packaging Elements Attention Analysis

The comparison between AOI with similar content in the packages allowed the authors to identify differences between the analyzed stimuli. The first attention (Table 4) was registered more quickly in the game image, with that of the Diset toy (D07, distributed in eight of the nine quadrants) being the one that required the least time (D07-AOI 7, TFF = 0, 42; E07-AOI 7, TFF = 1.05), highlighting that this corresponds to the behavior of women as well, with the order of marks changing in the case of men (E07 before D07). Below, attention was directed toward the name of the game (AOI 6, which occupies the quadrants Q1, Q2, and Q3 in both brands) for the two packages (again Diset, D06, more quickly, but with a reversal of behavior among men and women).

**TABLE 4:** Time First Fixation (TFF) of the total of participants to each AOI.

	**Group**		**Men**		**Women**	
	**TFF average**		**TFF average**		**TFF average**	
**AOI**	**EDUCA**	**DISET**	**EDUCA**	**DISET**	**EDUCA**	**DISET**
AOI 1	7.63	3.22	6.80	2.95	8.32	3.43
AOI 2	5.67	9.68	3.99	10.99	7.10	8.78
AOI 3	12.82	9.04	11.74	7.11	13.90	10.27
AOI 4	9.30	6.50	9.25	7.46	9.33	5.75
AOI 5	5.50	6.38	3.39	6.02	7.12	6.66
AOI 6	2.68	2.42	3.69	2.76	1.76	2.21
AOI 7	1.05	0.42	0.11	0.68	1.84	0.21

**Separation by gender. Source: Prepared by the authors.**

Both packages showed similar results regarding the stimulus (AOI 7) with greater attention in terms of the total fixation duration (Table 5) and the number of fixation counts (Table 6), with Diset having the greater prominence (D7-AOI 7, TFD = 16.21, FC = 52.08; E7-AOI 7, TFD = 7.85, FC = 25.54). Both men and women reflect the same behavior. However, the second stimulus that generates the longest total fixation duration for Diset is the number of questions (D01-AOI 1, TFD = 3.82; FC = 11.70), and for Educa the explanatory drawing of the topics (E02-AOI 2, TFD = 5.04; FC = 15.17), both represented by graphic elements, compared to the rest of the stimuli, which have a more textual design.

**TABLE 5:** Total Fixation Duration (TFD) of the total number of participants in each AOI.

	**Group**		**Men**		**Women**	
	**TFD average**		**TFD average**		**TFD average**	
**AOI**	**EDUCA**	**DISET**	**EDUCA**	**DISET**	**EDUCA**	**DISET**
AOI 1	1.79	3.82	5.33	12.01	1.95	3.99
AOI 2	5.04	1.24	15.28	3.38	5.43	1.39
AOI 3	0.44	0.59	1.63	1.67	0.39	0.65
AOI 4	0.68	2.08	3.64	6.80	0.45	2.12
AOI 5	0.73	1.24	3.07	4.63	0.59	1.13
AOI 6	2.00	1.56	7.49	6.23	1.77	1.36
AOI 7	7.85	16.21	28.41	55.85	7.28	15.78

**Separation by gender. Source: Prepared by the authors.**

**TABLE 6:** Fixation Count (FC) of the total of participants to each AOI.

	**Group**		**Men**		**Women**	
	**FC average**		**FC average**		**FC average**	
**AOI**	**EDUCA**	**DISET**	**EDUCA**	**DISET**	**EDUCA**	**DISET**
AOI 1	6.09	11.70	5.90	11.10	6.25	12.15
AOI 2	15.17	3.77	15.55	3.56	14.85	3.92
AOI 3	2.18	2.94	2.46	2.86	1.91	3.00
AOI 4	2.79	6.04	3.60	6.09	2.33	6.00
AOI 5	4.39	6.04	5.10	6.30	3.85	5.85
AOI 6	10.48	7.70	11.27	8.00	9.75	7.50
AOI 7	25.54	52.08	28.46	52.00	23.08	52.14

**Separation by gender. Source: Prepared by the authors.**

The previous data revealed that both men and women registered the same behavior regarding attention and fixations, so that the stimuli have the same effect, regardless of whether the group is made up of men or women.

### Analysis of Emotional Intensity

At the end of the biometry part, an in-depth semi-structured interview was carried out, recording the electrodermal activity. The questions were structured in three parts:

#### SECTION 1: Purchase Process

Q1. With what intention do you usually buy educational toys? (For your child, to give away, another).
Q2. Indicate, in order of priority, the criteria that you would most take into account when purchasing an educational toy (RECOMMENDED AGE, THEME, PACKAGE MESSAGES; OTHERS).

#### SECTION 2: Natural Memory Questions

Q3. Is there a concept that has caught your attention?
Q4. Do you remember which two brands were behind the games?
Q5. Do you remember the names of the toys that have been shown?
Q6. In which of the two brands do you perceive greater brand value? Why?
Q7. In which of the two brands do you perceive greater educational value? Why?

#### SECTION 3: Perception of Educational Value Through Packaging

The two packages are shown to the consumer.

Q8. What design (graphic) of the packaging is the most attractive to you? Why?
Q9. Through the packaging, which of the two toys has the greatest educational value? why?
Q10. Based on what the package transmits, for which of the two games would you pay more? Why?

##### SECTION 4: Final Choice

Q11. Taking into account the purchase intention and knowing the sale prices (12.95 euros Educa and 18.95 euros Diset), which one would you choose?

The most outstanding and frequent contributions from consumers are:

Q1: The prevailing purchase intention is “For your child,” along with “To give away.”
Q2: The recommended age and theme are key in choosing an educational toy, in that order.
Q3: It is intended to learn English, the recommended age, the Diset toy has more themes and more questions, Educa explains the themes better and they seem like the same game.
Q4: Most remember Educa, from their childhood. They have a harder time remembering the competition (Diset).
Q5: They don’t remember the name of the game. Some consumer remembers it in a distorted way.
Q6: Mainly they refer to Educa, for two reasons: the brand name and the experience lived in childhood.
Q7: Several consumers agree that Diset provides more value because the use of the game is clearer, the number of questions is greater and it seems less monotonous than that of Educa.
Q8: Diset transmits higher quality and is more colorful, it is easier to identify if they had to see it again on the shelf.
Q9: Similar designs. Diset seems more complete.
Q10: Diset, due to a greater availability of topics and questions, added to a higher quality perception.
Q11: If it is “for your child,” that of Educa. If it is “to give away,” Diset’s (greater social recognition and fewer problems if they need to return it).

These questions were asked to all the participants at the end of the experimental part, while the galvanic response of the skin was monitored. The interview, carried out under laboratory conditions, was intended to contrast certain aspects of the experimental part, without entering into a wider study. It was always the same person who asked the questions, and there was no time limit. The interview was conducted, in a semi-structured way. GSR values are relative. The objective was to locate the body changes that reflect the emotional state or somatic markers (Damásio, 1994). The somatic marker facilitates and speeds up decision-making, especially in social behavior, where situations of greater uncertainty can occur (Martínez Selva et al., 2006).

**FIGURE 4:**
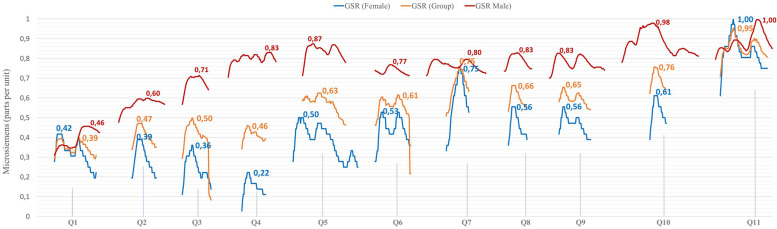
GSR peaks for each question. Group and by gender. Source: Prepared by the authors.

The GSR peaks (Figure 4) showed the highest emotional intensity (arousal) when expressing which toy they would choose, according to the reason for purchase, knowing the price (Q11). Social pressure (in the case of buying the toy with the intention of giving it away to a third party) determines that price is the key factor (the more expensive, the better), since it fits the budget and looks better. However, when it is for your own child, it is the educational factors (similar in perception through the packaging of both toys) and a lower price in the Educa brand, that opt for the Conector product. There is no difference between men and women (similar emotional arousal). In general terms (the values are expressed as one), there is a higher emotional level in the group of women, as they are the ones who usually buy the product (alone or accompanied by their partners). There is another peak of emotional intensity in which the levels also coincide between men and women and it is related to the question of which product has greater educational value (Q7), since there is a cognitive implication when trying to explain the choice with objective reasons.

## Discussion

The heat maps produced by the attention of the participants on the different parts of the packaging, for both brands (Figures 2, 3), qualitatively reflect more intense attention on the graphic elements, and so support the possible conclusion that graphic elements attract more attention (Orth et al., 2010). Moreover, the information on the number of questions, and to a lesser extent, on the brands that advertised or the name of the game, corroborate that packaging attributes are key in the purchase decision (Silayoi and Speece, 2007).

In the first comprehensive quantitative analysis of the attention that the entire group of subjects showed toward stimuli, it was observed that the image of the game attracted the higher percentage of attention of all participants, with no difference between genders. It is worth highlighting that the greater the area this stimulus occupies in the container, the greater the percentage of attention. The Diset image occupies 70% more area than that of Educa and is divided into eight quadrants, compared to the four quadrants of Educa), showing a significant effect of packaging elements design on product attention (Schoormans and Robben, 1997). The messages with a graphic component are the second in percentage of attention (game themes for Educa and operating message for Diset), generating the highest overall percentage. The number of questions (important anchor for parents, when quantifying the educational potential of the game) reflects a high percentage of attention. The designs with graphic shapes (Diset is designed in a round shape, compared to that of Educa, which is rectangular, apart from being 30% larger), again generates a higher percentage of attention.

Regarding the integral analysis of attention (Vecchiato and Roveda, 2010), the first attention is directed to the image of the game. Next, attention was drawn to the name of the game. However, the second stimulus that generates the most total duration of attention is the number of questions for Diset, and the explanatory drawing of the themes for Educa, both represented by graphic elements (Reimann et al., 2010), compared to the other stimuli, which have a more textual design. The previous data showed that both men and women registered the same behavior regarding attention and fixations, so that the stimuli have the same effect, regardless of whether the group is composed of men or women.

The areas of interest analyzed show the focused attention of consumers based on data and statistics (Zhang et al., 2018). These focuses of attention (main areas of interest displayed) have been endorsed by consumers through a qualitative study (in-depth interviews), who are concerned about or serve as decisive elements in the purchasing process. The areas of interest analyzed that concentrate the highest percentage of attention (the cover image of the game, the number of questions and topics, as well as the recommended age) are decisive in the purchase (Orquin and Loose, 2013), since they transmit the child’s experience, entertainment and expected level of learning. This approach must be complemented by the conclusions obtained in the in-depth interviews, which highlight a key insight when the product is going to be a gift: the price.

On the other hand, the performance of a qualitative study, monitored by GSR (neuro-qualitative) through an in-depth semi-structured interview, determines that the prevailing purchase intention is “For your child,” along with “To give away.” For this reason, consumers often buy educational toys for their children’s consumption, although they are also bought to make gifts.

The messages with a graphic component attracted the higher percentage of attention of all participants. Participants registered the same behavior regarding attention and fixations, so that the stimuli have the same effect, regardless of whether the group is composed of men or women. Only the qualitative study showed differences regarding the memory of the brands, being slightly less on the part of the mothers than of the fathers, in the case of Educa, which is the best positioned. It can be concluded that gender does not affect this type of educational toy.

This makes educational toys a concern for the development of the child, both his own, and family, and friends. The recommended age and theme are key in choosing an educational toy, in that order. The best remembered aspects are: it is intended to learn English, the recommended age and that the Diset toy has more themes and more questions. Most remember Educa, from their childhood. They have a harder time remembering the competition (Diset). They do not remember the name of the game (CONECTOR or LECTRON) although it has a lot of prominence on the cover. However, they do remember the theme (I learn English) and although they consider that both are brands of similar qualities, the majority indicates that Educa seems to them to have more value because they know it from childhood. The greatest perception of educational value is relative to the Educa brand, for two reasons: the brand name (Brunner et al., 2009) and the experience lived in childhood.

In general, the parents’ perception is that the designs are similar and transmit similar educational levels, but Diset’s seems more complete at the level of details aimed at the child and that of Educa aimed at a higher level of knowledge and focused on learning. The greater number of topics and questions in Diset leads the consumer to be willing to pay more, together with a perception of higher quality. This type of toy is more a gift to look good (gift to the son of a relative or a friend) or due to the concern of grandparents (gift to grandchildren), because there are already similar games for tablets (Cuesta-Cambra et al., 2017), something the child has in his hands every day.

The analysis of the GSR peaks showed a higher emotional intensity (arousal) when expressing which toy they would choose, according to the reason for purchase, knowing the price (Martínez-Ruiz et al., 2017). Social pressure (in the case of buying the toy with the intention of giving it away to a third party) determines that price is the key factor (the more expensive, the better), since it creates more prestige for the giver. However, when it is for your own child, it is the educational factors (similar in perception through the packaging of both toys) and a lower price in the Educa brand, that lead to consumers opting for the Conector product. There is no difference between men and women (similar emotional arousal). In general terms (the values are expressed as one), there is a higher emotional level in the group of women, as they are the ones who usually buy the product (alone or accompanied by their partners). There is another peak of emotional intensity in which the levels also coincide between men and women and it is relative to the question of which product has greater educational value, since there is a cognitive implication when trying to explain the choice with objective reasons (Lăzăroiu et al., 2017).

When the purchase reason is to give as a gift, notes that the Diset option is more “correct,” since the price is higher (approximate price of 16–18 euros, compared to Educa, whose approximate price is 10–12 euros). It has a background of social recognition, which leads the consumer to discard that of Educa, for being cheaper.

## Conclusion

The main objective of this study was to determine the degree of effectiveness of the packaging of an educational toy with the target audience and the sensations and emotions caused by the consumer (target) in the purchasing process (Fugate, 2008). This study has revealed the suggested buying and consumption habits (Calvert and Brammer, 2012) of educational toys, the most valued aspects in the consumption of educational toys, levels of brand and product recall, perception of brand and product value through packaging (Underwood and Ozanne, 1998), projection on the entertainment of toys from packaging (Basso et al., 2016) (design, size, shape, color, touch, etc.). It has also allowed the authors to compare the perception/coding of each container by men and women, identify the level of visual attraction (dedicated times) toward the product and the brand for the two packaging shown (average exposure times in the areas of interest, route, etc.) and analyze the levels of educational value of each container, perceived by the target customer (Enax et al., 2015).

This research has contributed to the change taking place in the scientific literature regarding the design of educational toy packaging. The recommendations drawn from the research regarding the design of educational toy packaging are aimed at graphically enhancing (Baudisch et al., 2003) those elements that really attract consumer attention: image of the game, number of questions/topics, brand, and recommended age. The authors consider it necessary to cover other types of educational toys, in order to generalize the results. The exploratory nature of experiment prevents generalization of the research results to other cases.

The analysis draws several conclusions that will help improve the perception of the toy, such as increasing the size of the brand, to improve educational positioning, at the cost of reducing other elements that generate less interest, such as the size of the product name. Similarly, using a graphic explanation of the game’s themes and increase the number of themes and questions (feeling of higher quality), as well as using brighter, warmer colors make the product more attractive. Furthermore, it is important to focus attention and explain the skills enhanced by the child playing, the age at which it is recommended to use and remove the remaining texts used (place them elsewhere other than the cover).

Finally, price can be key, depending on the purchase intention. The price influences the perception of greater educational and entertainment value. In turn, the intention to buy as a gift encourages the consumer to choose the one with the highest price, regardless of prior knowledge of the product or brand.

## Data Availability Statement

The raw data supporting the conclusions of this article will be made available by the authors, without undue reservation, to any qualified researcher.

## Ethics Statement

This study involving human participants was reviewed and approved by the Research Ethics Committee of the Universitat Politècnica de València (UPV). All participants gave their written informed consent, in accordance with the national legislation and the institutional requirements. Subjects were informed of their voluntary participation and anonymous contribution, as well as the possibility of withdrawing from the study at any time without reason. Written, informed consent was obtained from the brand EDUCA for the publication of any potentially identifiable images or data included in this article.

## Author Contributions

DJ conceived, designed, and performed the experiments, contributed reagents, materials, analysis tools, and data, and wrote the manuscript. VT-V and AM analyzed and interpreted the data, contributed reagents, materials, analysis tools, and data, and wrote the manuscript. All authors contributed to the article and approved the submitted version.

## Conflict of Interest

The authors declare that the research was conducted in the absence of any commercial or financial relationships that could be construed as a potential conflict of interest.
